# ﻿*Paramphibambusabambusicola* gen. et. sp. nov., *Arecophilaxishuangbannaensis* and *A.zhaotongensis* spp. nov. in Cainiaceae from Yunnan, China

**DOI:** 10.3897/mycokeys.104.117872

**Published:** 2024-04-16

**Authors:** Li-Su Han, Nalin N. Wijayawardene, Chao Liu, Li-Hong Han, Itthayakorn Promputtha, Qiang Li, Abdallah M. Elgorban, Salim Al-Rejaie, Kazuaki Tanaka, Dong-Qin Dai

**Affiliations:** 1 Center for Yunnan Plateau Biological Resources Protection and Utilization, College of Biological Resource and Food Engineering, Qujing Normal University, Qujing, Yunnan 655011, China; 2 Tropical Microbiology Research Foundation, 96/N/10, Meemanagoda Road, 10230 Pannipitiya, Sri Lanka; 3 Department of Biology, Faculty of Science, Chiang Mai University, Chiang Mai 50200, Thailand; 4 Department of Botany and Microbiology, College of Science, King Saud University, Riyadh, Saudi Arabia; 5 Department of Pharmacology & Toxicology, College of Pharmacy, King Saud University, Riyadh, Saudi Arabia; 6 Faculty of Agriculture and Life Science, Hirosaki University, Bunkyo-cho 3, Hirosaki, Aomori 036-8561, Japan

**Keywords:** Bambusicolous fungi, multilocus phylogeny, taxonomy, Xylariales

## Abstract

Morphological comparisons and multi locus phylogenetic analyses (base on the combined genes of ITS, LSU, *rpb*2 and *tub*) demonstrated that three new saprobic taxa isolated from bamboo belong to Cainiaceae. These taxa comprise a novel genus *Paramphibambusa* (*P.bambusicola***sp. nov.**) and two new species, *Arecophilaxishuangbannaensis* and *A.zhaotongensis*. The three new taxa belong to Cainiaceae (Xylariales, Sordariomycetes) a poorly studied family, which now comprises eight genera. *Paramphibambusa* can be distinguished from other Cainiaceae genera in having ascomata with a neck and ascospores lacking longitudinal striation, germ slits or germ pores. The two new *Arecophila* species clustered in a clade with *Arecophila* sp. and *A.bambusae*. Detailed morphological descriptions, illustrations, and an updated phylogenetic tree are provided for the new taxa.

## ﻿Introduction

During our continuous investigation of bambusicolous fungi in Yunnan, China, we have collected one new genus and two new *Arecophila* K.D. Hyde species in Cainiaceae J.C. Krug. The family Cainiaceae (Xylariales, Sordariomycetes) was established by Krug (1978), with *Cainia* Arx & E. Müll as the type genus. Hongsanan et al. (2017) and Wijayawardene et al. (2020) accommodated Cainiaceae in Xylariomycetidae family *incertae sedis*. However, Hyde et al. (2020), Samarakoon et al. (2021), and Wijayawardene et al. (2022a) accepted Cainiaceae in Xylariales.

Maharachchikumbura et al. (2015, 2016) accepted five genera (*viz.*, *Amphibambusa* D.Q. Dai & K.D. Hyde, *Arecophila*, *Atrotorquata* Kohlm. & Volkm.-Kohlm, *Cainia*, and *Seynesia* Sacc.) in Cainiaceae based on morphology and phylogeny. Subsequently, Mapook et al. (2020) introduced *Longiappendispora* Mapook & K.D. Hyde as a new member of Cainiaceae. Konta et al. (2021) transferred *Endocalyx* Berk. & Broome from Apiosporaceae to Cainiaceae. Li et al. (2022) revisited the monospecific genus *Alishanica* Karun et al. and synonymized it under *Arecophila*. Hence, seven genera (*Amphibambusa*, *Arecophila*, *Atrotorquata*, *Cainia*, *Endocalyx*, *Longiappendispora*, *Seynesia*) are accepted in Cainiaceae according to Hyde et al. (2020), Mapook et al. (2020), Jiang et al. (2021), and Konta et al. (2021).

Members of Cainiaceae are often found in tropical and temperate regions as saprobic fungi, which are usually associated with monocotyledons (mainly grasses) and fabaceous dicotyledons. Some *Cainia* species have been reported as causative agents of plant diseases, e.g., *C.desmazieri* C. Moreau & E. Müll (Krug 1978). Cainiaceae is morphologically characterized by immersed ascomata with a papillate ostiole, unitunicate asci, with a unique J+, apical ring or series of rings, and hyaline to pigmented, 1-septate ascospores with longitudinal striations or germ slits or germ pores, and usually surrounded by a sheath or appendages (Maharachchikumbura et al. 2016; Hyde et al. 2020). Asexual morphs of this family were reported as coelomycetous taxa, *viz.*, *Cainia* and *Endocalyx*, that are characterized by black, pycnidial conidiomata, denticulate, sympodially proliferating conidiophores, branched or simple, septate, and phialidic conidiogenous cells, and hyaline, fusiform, or falcate to lunate conidia (Maharachchikumbura et al. 2016; Hyde et al. 2020; Konta et al. 2021; Wijayawardene et al. 2021a).

*Arecophila* was introduced by Hyde (1996) with *A.gulubiicola* K.D. Hyde as the type species. The genus *Arecophila* was initially regarded as a member of Amphisphaeriaceae G. Winter based on the morphology. Subsequently, Kang et al. (1999) accepted *Arecophila* as a member of Cainiaceae. Afterwards, the placement of *Arecophila* within the Cainiaceae has been confirmed based on analyses of partial LSU gene sequences (Jeewon et al. 2003; Senanayake et al. 2015; Li et al. 2022). Currently, 18 epithets are listed under *Arecophila* based on morpho-molecular study (Li et al. 2022; Index Fungorum 2023), and 15 epithets are listed under *Arecophila* in Species Fungorum (2023).

According to Jiang et al. (2022) and previous studies (Eriksson and Yue 1998; Hyde et al. 2002a, b; Zhou and Hyde 2002; Cai et al. 2003), only four Cainiaceae species are associated with bamboo (*Amphibambusahongheensis* H.B. Jiang & Phookamsak, *Arecophilabambusae* Umali & K.D. Hyde, *A.coronata* (Rehm) Umali & K.D. Hyde and *A.nypae* K.D. Hyde) in China. In this study, we aim to collect bamboo samples in Yunnan, China, describe and introduce a new genus *Paramphibambusa* to accommodate *P.bambusicola*, and two new species *Arecophilaxishuangbannaensis* and *A.zhaotongensis* in the family of Cainiaceae. This study enriches the species diversity of bambusicolous Cainiaceae species in China.

## ﻿Materials and methods

### ﻿Sample collection, single spore isolation and morphological study

Bamboo culms were collected in northeastern (Zhaotong), northwestern (Shangri-La), and southwestern (Xishuangbanna) Yunnan Province, China, stored in disposable plastic Ziplock bags and brought back to the laboratory for examination and study. Morphological observation and single spore isolation were followed as described in Dai et al. (2017). The ascomata on the host surface were observed by Leica using a S8AP0 microscope and photographed by HDMI 200C. Micro-morphological features were observed using an Olympus BX53 compound microscope and captured with an Olympus DP74 camera (Olympus SZ61; Olympus Corporation, Tokyo, Japan). The asci were stained by Meltzer’s reagent to examine the J-/J+ ring at the tip of the asci. India ink was used to stain the ascospores for checking the mucilaginous sheath. The micro-morphological features and fruiting bodies were measured by Tarosoft (R) Image FrameWork (IFW). The photo plates were created by Adobe Photoshop CS6 software (Adobe Systems Inc., San Jose, CA, USA). Herbarium material and living cultures were deposited at the Herbarium of Guizhou Medical University (GMB), Guizhou Medical University Culture Collection (GMBCC) Guiyang, Zhongkai University of Agriculture and Engineering (ZHKU), Zhongkai University of Agriculture and Engineering Culture Collection (ZHKUCC) Guangdong, China, and the Guizhou Culture Collection (GZCC), Guiyang, China. MycoBank numbers were obtained from MycoBank database (https://www.mycobank.org/; accessed on 23 January 2024) to register the newly described taxa (MycoBank 2024).

### ﻿DNA extraction, PCR amplification and sequencing

Fungal genomic DNA was extracted from fresh mycelium using the Biospin Fungus Genomic DNA Extraction Kit (BioFlux) according to the manufacturer’s instructions. When culture could not be obtained, fruiting bodies were used to extract genomic DNA by using E.Z.N.A. Forensic DNA Kit (BIO-TEK) followed the protocols. Genomic DNA was conducted by polymerase chain reaction (PCR). Four phylogenetic markers, internal transcribed spacer (ITS), large-subunit ribosomal RNA (LSU), RNA polymerase II (*rpb*2), and *tub*, were amplified using primer pairs ITS4/ITS5 (White et al. 1990), LR5/LR0R (Vilgalys and Hester 1990), RPB2-5F/RPB2-7cR (Liu et al. 1999), Bt2a/Bt2b (Hsieh et al. 2005), respectively. Amplification conditions were performed according to Dai et al. (2022) and Li et al. (2022). The purified PCR fragments were sequenced at Shanghai Myobio Biomedical Technology Co. and China UW Genetics Solutions (BGI-Tech), in Shanghai, China. The newly obtained sequence data were deposited in GenBank (https://www.ncbi.nlm.nih.gov).

### ﻿Sequence alignment and phylogenetic analyses

The newly generated reverse and forward sequences were assembled with Geneious (Restricted) 9.1.2 (https://www.geneious.com, accessed on 20 May 2023) and subjected to BLAST searches in GenBank (https://blast.ncbi.nlm.nih.gov/, accessed on 20 May 2023) for revealing closely matched strains (Table 1). The related sequences of families in the order Xylariales were downloaded based on the latest article Li et al. (2022). The single gene matrix was aligned via the server version of MAFFT v. 7 (Katoh and Standley 2013) (https://mafft.cbrc.jp/alignment/server). The aligned sequence datasets were trimmed by trimAl.v1.2rev59. The alignments were combined via SequenceMatrix 1.9 (Vaidya et al. 2011). The AliView 1.26 (Larsson 2014) was used to obtain phylip and nexus format files for RAxML analysis and Bayesian analysis, respectively.

**Table 1.** Sequences used for phylogenetic analyses in this study. The newly generated sequences are in bold. Type strains or type specimens are labelled with HT (holotype), ET (epitype), IT (isotype), and PT (paratype), T (Type), “N/A” indicates no available sequences.

Species	Strain/voucher No.	Status	GenBank accession numbers			
			ITS	LSU	*rpb*2	*tub*
*Amphibambusabambusicola*	MFLUCC 11-0617	HT	KP744433	KP744474	NA	NA
*Amphibambusahongheensis*	KUN-HKAS 112723	HT	MW892971	MW892969	NA	NA
*Amphibambusahongheensis*	KUMCC 20-0334	HT	MW892972	MW892970	NA	NA
*Amphiroselliniafushanensis*	HAST 91111209	HT	GU339496	NA	GQ848339	GQ495950
*Amphirosellinianigrospora*	HAST 91092308	HT	GU322457	NA	GQ848340	GQ495951
*Annulohypoxylonatroroseum*	ATCC 76081	–	AJ390397	KY610422	KY624233	DQ840083
*Annulohypoxylonstygium*	MUCL 54601	–	KY610409	KY610475	KY624292	KX271263
*Apiosporaarundinis*	CBS 464.83	–	KF144888	KF144933	NA	KF144979
*Apiosporahysteriana*	ICMP 6889	–	NA	DQ368630	DQ368649	DQ368621
*Apiosporakogelbergense*	CBS 117206	–	KF144895	KF144941	NA	KF144987
*Apiosporasetosa*	ATCC 58184	–	NA	AY346259	NA	NA
*Arecophilaaustralis*	GZUCC0112	HT	MT742126	MT742133	NA	MT741734
*Arecophilaaustralis*	GZUCC0124	PT	MT742125	MT742132	NA	NA
*Arecophilabambusae*	HKUCC 4794	–	NA	AF452038	NA	NA
*Arecophilaclypeata*	GZUCC0110	HT	MT742129	MT742136	MT741732	NA
*Arecophilaclypeata*	GZUCC0127	PT	MT742128	MT742135	NA	NA
*Arecophilamiscanthi*	GZUCC0122	–	MT742127	MT742134	NA	NA
*Arecophilamiscanthi*	MFLU 19-2333	HT	NR_171235	MK503827	NA	NA
*Arecophila* sp.	HKUCC 6487	–	NA	AF452039	NA	NA
***Arecophilaxishuangbannaensis***	**ZHKU 23-0280**	–	**OR995737**	**OR995744**	**NA**	**NA**
***Arecophilaxishuangbannaensis***	**GMB-W1283**	**HT**	**OR995736**	**OR995743**	**NA**	**NA**
***Arecophilazhaotongensis***	**GMBCC1145**	**HT**	**OR995740**	**OR995747**	**OR995741**	**NA**
***Arecophilazhaotongensis***	**ZHKU 23-0260**	–	**OR995738**	**OR995745**	**NA**	**NA**
***Arecophilazhaotongensis***	**ZHKU 23-0259**	**IT**	**OR995735**	**OR995742**	**NA**	**NA**
*Astrocystisconcavispora*	MFLUCC 14-0174	HT	KP297404	KP340545	KP340532	KP406615
*Atrotorquatalineata*	HKUCC 3263	–	AF009807	NA	NA	NA
*Atrotorquataspartii*	MFLUCC 13-0444	HT	NA	KP325443	NA	NA
*Barrmaeliarappazii*	CBS 142771	HT	MF488989	MF488989	MF488998	MF489017
*Barrmaeliarhamnicola*	CBS 142772	ET	MF488990	MF488990	MF488999	MF489018
*Cainiaanthoxanthis*	MFLUCC 15-0539	HT	NR_138407	KR092777	NA	NA
*Cainiadesmazieri*	CAI	–	KT949896	KT949896	NA	NA
*Cainiadesmazieri*	CBS 137.62	–	MH858124	MH869702	NA	NA
*Cainiaglobosa*	MFLUCC 13-0663	HT	NR_171724	KX822123	NA	NA
*Cainiagraminis*	CBS 136.62	–	MH858123	AF431949	NA	NA
*Cainiagraminis*	MFLUCC 15-0540	–	KR092793	KR092781	NA	NA
*Cainia* sp.	LSU0560	–	MT000421	MT000513	NA	NA
*Camilleaobularia*	ATCC 28093	–	KY610384	KY610429	KY624238	KX271243
*Camilleatinctor*	YMJ 363	–	JX507806	NA	JX507790	JX507795
*Collodisculabambusae*	GZ 62	–	KP054279	KP054280	KP276675	KP276674
*Collodisculafangjingshanensis*	GZUH 0109	HT	KR002590	KR002591	KR002592	KR002589
*Coniocessiamaxima*	CBS 593.74	HT	NR_137751	MH878275	NA	NA
*Coniocessianodulisporioides*	CBS 281.77	IT	MH861061	AJ875224	NA	NA
*Creosphaeriasassafras*	STMA 14087	–	KY610411	KY610468	KY624265	KX271258
*Daldiniabambusicola*	CBS 122872	HT	KY610385	KY610431	KY624241	AY951688
*Daldiniaconcentrica*	CBS 113277	–	AY616683	KT281895	KY624243	KC977274
*Endocalyxcinctus*	NBRC 31306	–	MZ313191	MZ313152	NA	NA
*Endocalyxcinctus*	JCM 7946	–	LC228648	LC228704	NA	NA
*Endocalyxgrossus*	JCM 5164	HT	MZ313160	MZ313138	NA	NA
*Endocalyxgrossus*	JCM 5165	–	MZ313159	MZ313158	NA	NA
*Endocalyxgrossus*	JCM 5166	–	MZ313179	MZ313171	NA	NA
*Endocalyxindumentum*	JCM 5171	HT	MZ313153	MZ313161	NA	NA
*Endocalyxindumentum*	JCM 8042	–	MZ313162	MZ313157	NA	NA
*Endocalyxmelanoxanthus*	CBS147393	–	MW718204	MW718204	NA	NA
*Endocalyxmelanoxanthus*	CBS147394	–	MW718203	MW718203	NA	NA
*Endocalyxptychospermatis*	ZHKUCC 21-0008	HT	MZ493352	OK513439	NA	NA
*Endocalyxptychospermatis*	ZHKUCC 21-0009	HT	MZ493353	OK513440	NA	NA
*Endocalyxptychospermatis*	ZHKUCC 21-0010	HT	MZ493354	OK513441	NA	NA
*Entoleucamammata*	JDR 100	–	GU300072	NA	GQ844782	GQ470230
*Entonaemaliquescens*	ATCC 46302	–	KY610389	KY610443	KY624253	KX271248
*Entosordariaperfidiosa*	CBS 142773	ET	MF488993	MF488993	MF489003	MF489021
*Entosordariaquercina*	RQ/CBS 142774	HT	MF488994	MF488994	MF489004	MF489022
*Graphostromaplatystomum*	CBS 270.87	HT	JX658535	AY083827	KY624296	HG934108
*Hypocoprarostrata*	NRRL 66178	–	KM067909	KM067909	NA	NA
*Hypocreagelatinosa*	NBRC 104900	ET	JN943358	JN941453	NA	NA
*Hypomontagnellabarbarensis*	STMA 14081	HT	MK131720	MK131718	MK135891	MK135893
*Hypomontagnellamonticulosa*	MUCL 54604	ET	KY610404	KY610487	KY624305	KX271273
*Hypoxylonfragiforme*	MUCL51264	ET	KM186294	KM186295	KM186296	KX271282
*Hypoxyloninvestiens*	CBS 118185	–	KC968924	KY610451	KY624260	KC977269
*Jackrogersellamultiformis*	CBS 119016	ET	KC477234	KT281893	KY624290	KX271262
*Kretzschmariadeusta*	CBS 163.93	–	KC477237	KY610458	KY624227	KX271251
*Leiosphaerellachromolaenae*	CBS 125586	–	JF440976	JF440976		
*Longiappendisporachromolaenae*	MFLUCC 17-1485	HT	NR_169723	NG_068714	NA	NA
*Lopadostomaamericanum*	LG8	HT	KC774568	KC774568	KC774525	NA
*Lopadostomadryophilum*	LG21	ET	KC774570	KC774570	KC774526	MF489023
*Lopadostomafagi*	LF1	HT	KC774575	KC774574	KC774531	NA
*Lopadostomaquercicola*	LG27	HT	KC774610	KC774610	KC774558	NA
*Lopadostomaturgidum*	LT2	ET	KC774618	KC774618	KC774563	MF489024
*Monographellanivalis*	UPSC 3273	–	NA	AF452030	NA	NA
*Nemaniaabortiva*	BISH 467	HT	GU292816	NA	GQ844768	GQ470219
*Nemaniabipapillata*	HAST 90080610	–	GU292818	NA	GQ844771	GQ470221
*Nemaniamaritima*	HAST 89120401	ET	GU292822	NA	GQ844775	GQ470225
*Nemaniaprimolutea*	HAST 91102001	HT	EF026121	NA	GQ844767	EF025607
*Obolarinadryophila*	MUCL 49882	–	GQ428316	GQ428316	KY624284	GQ428322
*Oxydothisfrondicola*	HKUCC 1001	–	NA	AY083835	NA	NA
***Paramphibambusabambusicola***	**GMBCC1142**	**HT**	**OR995739**	**OR995746**	**OR995740**	**NA**
***Paramphibambusabambusicola***	**ZHKUCC 23-0976**	–	**OR995741**	**OR995748**	**OR995739**	**NA**
*Paraxylariaxylostei*	MFLU 17-1636	–	MW240640	MW240570	NA	MW820914
*Paraxylariaxylostei*	MFLU 17-1645	–	MW240641	MW240571	NA	MW820915
*Phylaciasagrana*	CBS 119992	–	AM749919	NA	NA	NA
*Podosordariamexicana*	WSP 176	–	GU324762	NA	GQ853039	GQ844840
*Podosordariamuli*	WSP 167	HT	GU324761	NA	GQ853038	GQ844839
*Poroniapileiformis*	WSP 88113001	ET	GU324760	NA	GQ853037	GQ502720
*Poroniapunctata*	CBS 656.78	HT	KT281904	KY610496	KY624278	KX271281
*Pyrenopolyporusnicaraguensis*	CBS 117739	–	AM749922	KY610489	KY624307	KC977272
*Rhopalostromaangolense*	CBS 126414	–	KY610420	KY610459	KY624228	KX271277
*Roselliniaaquila*	MUCL 51703	–	KY610392	KY610460	KY624285	KX271253
*Roselliniacorticium*	MUCL 51693	–	KY610393	KY610461	KY624229	KX271254
*Rostrohypoxylonterebratum*	CBS 119137	HT	DQ631943	DQ840069	DQ631954	DQ840097
*Ruwenzoriapseudoannulata*	MUCL 51394	HT	KY610406	KY610494	KY624286	KX271278
*Sarcoxyloncompunctum*	CBS 359.61	–	KT281903	KY610462	KY624230	KX271255
*Seynesiaerumpens*	SMH 1291	–	NA	AF279410	AY641073	NA
*Stilbohypoxylonquisquiliarum*	YMJ 172	–	EF026119	NA	GQ853020	EF025605
*Thamnomycesdendroideus*	CBS 123578	–	FN428831	KY610467	KY624232	KY624313
*Vialaeamangiferae*	MFLUCC 12-0808	HT	KF724974	KF724975	NA	NA
*Vialaeaminutella*	BRIP 56959	–	KC181926	KC181924	NA	NA
*Xylariahypoxylon*	CBS 122620	ET	KY610407	KY610495	KY624231	KX271279
*Zygosporiumoscheoides*	MFLUCC 14-0402	–	MF621585	MF621589	NA	NA

Maximum likelihood (ML) analysis was performed by RAxML-HPC2 on XSEDE (8.2.12) (Stamatakis et al. 2008; Stamatakis 2014) via the CIPRES Science Gateway V.3.3 web server (https://www.phylo.org/portal2/login!input. action) (Miller et al. 2010). The best model was GTRGAMMA, with 1000 replicates rapid bootstrapping. Bayesian inference (BI) analysis was performed by MrBayes on XSEDE (3.2.7a) in the website CIPRES Science Gateway (Ronquist et al. 2012). Markov Chain Monte Carlo (MCMC) was used to evaluate posterior probabilities (PP) (Rannala and Yang 1996; Zhaxybayeva and Gogarten 2002). The best model test for each gene was performed via MrMTgui (Ma 2016). Six simultaneous Markov chains were run for 1000000 generations, and trees were sampled every 100^th^ generation (resulting in 10,000 total trees). The phylogenetic trees were visualized with FigTree v. 1.4.2 (http://tree.bio.ed.ac.uk software/figtree/) (Rambaut 2012), and edited by Adobe Illustrator CS v. 5.

### ﻿Abbreviations

**ATCC**: American Type Culture Collection; **BISH**: Bishop Museum, Department of Natural Sciences; **CAI**: Cairo University, Botany Department; **CBS**: Culture Collection of the Westerdijk Fungal Biodiversity Institute, Utrecht, Netherlands; **GMBCC**: Guizhou Medical University Culture Collection, Guiyang, China; **GZU**: Karl-Franzens-Universitat Graz; **GZUCC**: Guizhou University Culture Collection, Guiyang, Guizhou, China; **HAST**: Research Center for Biodiversity, Academia Sinica; **HKUCC**: The University of Hong Kong Culture Collection, Hong Kong, P.R. China; **JCM**: Japan Collection of Microorganisms, Japan; **JDR**: J.D. Rogers; **KUMCC**: Kunming Institute of Botany Culture Collection; **KUN-HKAS**: Herbarium of Cryptogams Kunming Institute of Botany Academia Sinica; **LF**: *LopadostomafagiL*; **LT**: *Lopadostomaturgidum*; **MFLU**: Mae Fah Luang University Herbarium; **MFLUCC**: Mae Fah Luang University Culture Collection; **MUCL**: Agro-food & Environmental Fungal Collection; **NBRC**: Biological Resource Center IFO; **NRRL**: Agricultural Research Service Culture Collection; **SMH**: Sabine M. Huhndorf; **KUN-HKAS**: Herbarium of Cryptogams Kunming Institute of Botany Academia Sinica; **STMA**: HZI culture collection, Helmholtz Centre for Infection Research, Braunschweig, Germany; **WSP**: Washington State University, Plant Pathology Department; **YMJ**: YuMing, Ju; **ZHKUCC**: Zhongkai University of Agriculture and Engineering.

## ﻿Results

### ﻿Phylogenetic results

The combined dataset comprised 107 strains (Table 1). *Hypocreagelatinosa* (NBRC 104900) was selected as the outgroup taxon. The alignment comprised 4195 bp in total (ITS 580 bp, LSU 736 bp, *rpb*2 1197 bp, and *tub* 1682 bp). The final ML optimization likelihood value of -68750.486429 and the matrix had 2603 bp distinct alignment patterns, with 45.50% of undetermined characters or gaps. Estimated base frequencies were as follows: A = 0.240607, C = 0.260776, G = 0.259542, T = 0.239075, AC = 1.358257, AG = 3.703167, AT = 1.354909, CG = 1.087664, CT = 6.069506, GT = 1.000000; proportion of invariable sites I = 0.378984; and gamma distribution shape parameter α = 0.817253.

**Figure 1.**
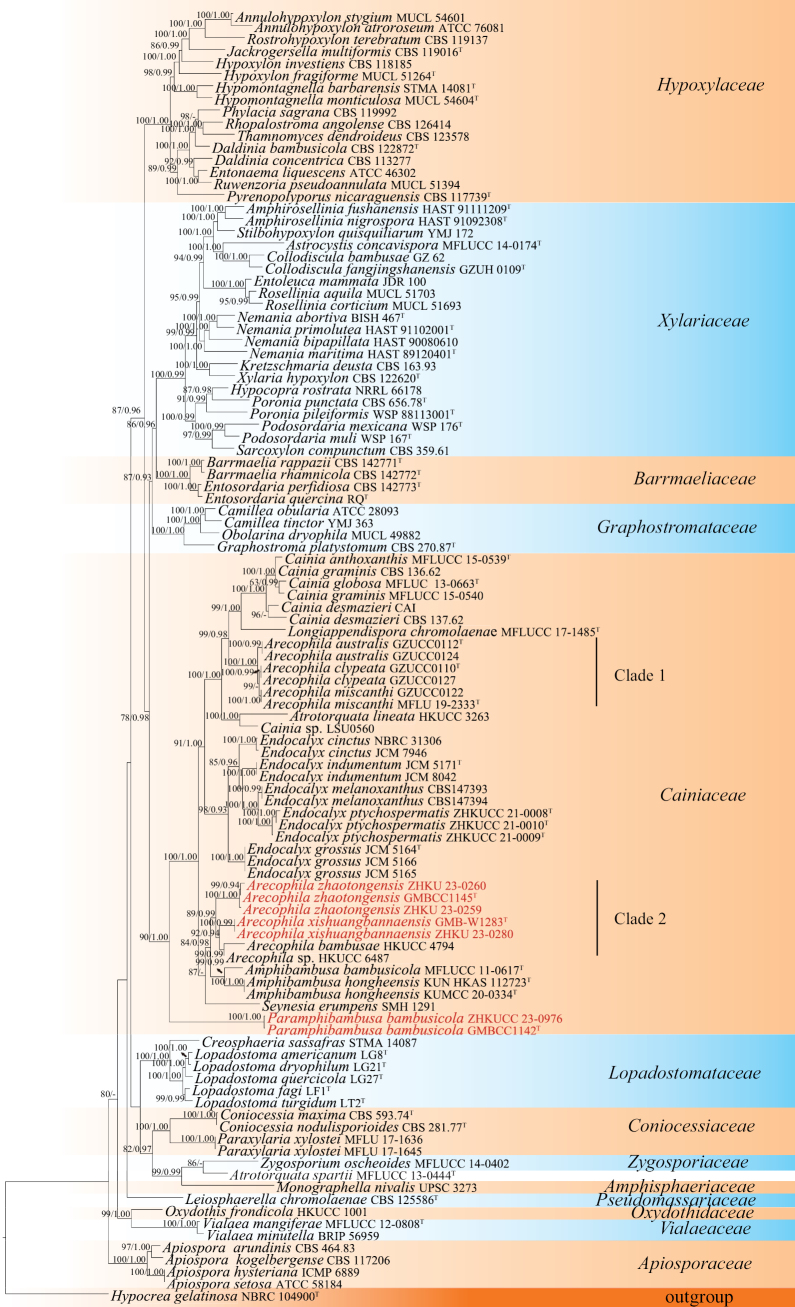
The RAxML tree was generated based on the combined ITS, LSU, *rpb*2, and *tub* sequence data. Bootstrap support values for ML equal to or greater than 60%, and Bayesian posterior probabilities (BYPP) equal to or higher than 0.90 are indicated above the nodes as ML/PP. Type materials are indicated by superscript “T”, while the newly generated sequences are shown in red.

The final RAxML tree (Fig. 1) is based on maximum likelihood (ML), and Bayesian inference analyses with similar topology. The RAxML tree showed that *Paramphibambusabambusicola* (GMBCC1142, ZHKUCC 23-0976) formed a distinct, stable clade basal to the other genera of Cainiaceae with high statistical support (90% ML, 1.00 PP). Moreover, *Arecophila* strains form two clades (Fig. 1), which coincide with Li et al. (2022). Our new collections cluster with *A.bambusae* Umali & K.D. Hyde (HKUCC 4794) and *Arecophila* sp. (HKUCC 6487) forming a sister branch clustered in Clade 2 (Fig. 1).

### ﻿Taxonomy

#### ﻿ Paramphibambusa

Taxon classificationFungiXylarialesCainiaceae

L.S. Han & D.Q. Dai gen. nov.

CD63B0CA-BDB9-5768-94F0-2CD297BA0F6A

MB851854

##### Etymology.

In reference to a new genus is morphologically similar to *Amphibambusa*, but phylogenetically distinct.

##### Description.

***Saprobic*** on bamboo culms. **Sexual morph: *Ascomata*** deeply immersed beneath poorly developed clypeus, solitary, scattered, black, globose to subglobose, ostiolate, with a long neck. ***Peridium*** composed of several layers, thick-walled, hyaline to pale brown cells of textura angularis. ***Paraphyses*** hyaline, numerous, filiform to cylindrical, guttulate, branched, septate, tapering towards the apex. ***Asci*** 8-spored, rarely 6-spored, unitunicate, cylindrical, short pedicellate, straight or slightly curved, rounded at the apex, with an elliptical to trapezoidal, J+ sub-apical ring. ***Ascospores*** uniseriate or overlapping uniseriate, hyaline to golden brown, ellipsoidal, guttulate, 2–3-celled, tapering at the ends, slightly constricted at the septum, smooth-walled, surrounded by a mucilaginous sheath. **Asexual morph**: Undetermined.

##### Type species.

*Paramphibambusabambusicola* L.S. Han & D.Q. Dai

##### Notes.

A monotypic genus *Paramphibambusa* is introduced based on its different morphological characteristics and the support of phylogenetic affinity with the other members in Cainiaceae. The morphological characteristics of *Paramphibambusa* resemble *Amphibambusa* in having dark clypeus, immersed, globose to subglobose ascomata, unitunicate, short pedicellate asci with a J+, and sub-apical ring, and 1-septate ascospores, surrounded by a thick mucilaginous sheath (Liu et al. 2015; Jiang et al. 2021). *Paramphibambusa* can be easily distinguished from *Amphibambusa* in having an ostiole, with a long neck, and ascospores lacking longitudinal wall ornamentations. In addition, *Paramphibambusa* forms a well-separated branch basal to other cainiaceous genera with 90% ML, and 1.00 PP statistical supports (Fig. 1). *Paramphibambusa* differs from the sexual members of Cainiaceae in ascomata with a long neck leading up to the ostiole, and in that the ascospores lack longitudinal striations or germ slits or germ pores *Endocalyx* is an asexually typified genus and lacks a sexual morph to compare its morphology with *Paramphibambusa*. However, in the phylogenetic analyses, *Paramphibambusa* resides in a distinct phylogenetic lineage to *Endocalyx* (Fig. 1). Therefore, we consider *Paramphibambusa* as a distinct genus.

#### ﻿ Paramphibambusa bambusicola

Taxon classificationFungiXylarialesCainiaceae

L.S. Han & D.Q. Dai sp. nov.

9B44C681-26B4-5A60-B4FF-8DC259F346B3

MB851857

Fig. 2

##### Etymology.

With reference to its occurrence on host bamboo.

##### Holotype.

GMB-W1350.

##### Description.

***Saprobic*** on dead culms of bamboo. **Sexual morph**: ***Ascomata*** 430–580 × 500–550 µm (x– = 474 × 519 µm, n = 20), deeply immersed beneath blackened poorly developed clypeus, solitary, scattered, black, globose to subglobose, ostiolate, with a long neck, 50–125 µm diam., 240–260 µm long. ***Peridium*** 15–25 µm thick, composed of several layers, thick-walled, hyaline to pale brown cells of textura angularis. ***Paraphyses*** 2–5.5 µm wide, hyaline, numerous, filiform to cylindrical, guttulate, branched, septate, tapering towards the apex. ***Asci*** 200–240 × 10–13.5 µm (x– = 215 × 11.5 µm, n = 20), 8-spored, rarely 6-spored, unitunicate, cylindrical, short pedicellate, straight or slightly curved, rounded at the apex, with a 3–4 µm wide, 1.5–2 µm high (x– = 3.6 × 1.7 µm, n = 20), elliptical to trapezoidal, J+, sub-apical ring. ***Ascospores*** 24–35 × 6–7.5 µm (x– = 27 × 6.6 µm, n = 20), uniseriate or overlapping uniseriate, hyaline to golden brown, ellipsoidal, 2–3-celled, tapering at the ends, slightly constricted at the septum, smooth-walled, surrounded by a 9–12 µm mucilaginous sheath. **Asexual morph**: Undetermined.

##### Culture characters.

Ascospores germinating within 24 h. Colonies reaching 45 mm diam. in 20 days under dark and at 28 °C conditions, circular, flocculent, yellowish from above and below.

**Figure 2.**
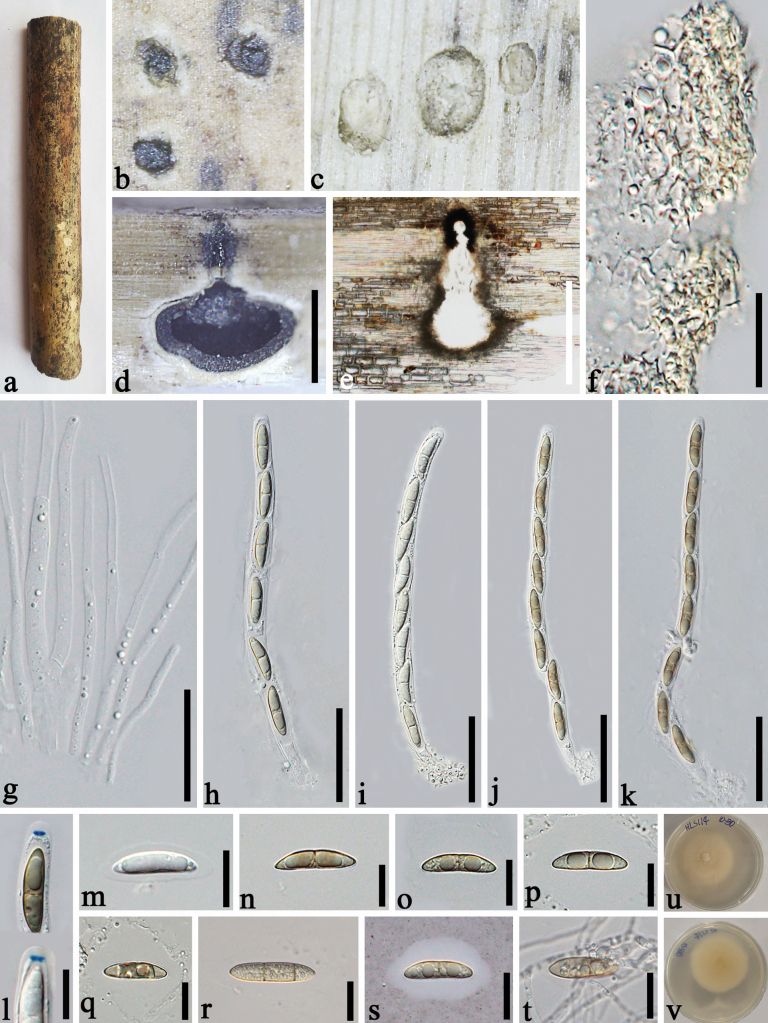
*Paramphibambusabambusicola* (GMB-W1350, holotype) **a** bamboo specimen **b** black ostioles at the host surface **c** transverse section of ascomata **d, e** vertical section of ascomata with long necks and black clypeus **f** cells of peridium **g** paraphyses **h–k** asci **l** asci with J+, elliptical to trapezoidal, subapical ring (stained in Melzer’s reagent) **m–s** ascospores (**s** ascospore stained in Indian ink showing mucilaginous sheath) **t** a germinating ascospore **u, v** cultures on PDA after 20 days (**u** upper, **v** reverse). Scale bars: 300 µm (**d, e**); 15 µm (**f, l–t**); 30 µm (**g**); 50 µm (**h–k**).

##### Materials examined.

China, Yunnan Province, Zhaotong, Zhenxiong town, 27°36′8"N, 104°56′34"E, 1673.07 m, on dead culms of bamboo, 29 July 2021, Dong-Qin Dai, Li-Su Han, DDQ02077, (GMB-W1350, holotype), GMBCC1142, ex-type; *ibid*. (ZHKU 23-0256, isotype), GZCC 23-0629, ex-isotype; Zhaotong, Zhenxiong town, Shanzhai, 27°62′52"N, 104°81′98"E, 1666.10 m, on dead culms of bamboo, 4 August 2023, Dong-Qin Dai, Li-Su Han, HLS0114 (ZHKU 23-0257), living culture ZHKUCC 23-0976.

##### Notes.

In the phylogenetic tree, *Paramphibambusabambusicola* formed a stable clade basal to the other species of Cainiaceae with 90% ML, and 1.00 PP statistical supports (Fig. 1). In morphology, *Paramphibambusabambusicola* has Cainiaceae species typical characteristics that are cylindrical asci, with a J+, apical ring, and ellipsoidal ascospores surrounded by a mucilaginous sheath. However, the spores of Cainiaceae species have the ornamented walls with longitudinal striations or germ slits or germ pores. *Paramphibambusabambusicola* differs from the current Cainiaceae species by having smooth-walled ascospores. Therefore, based on morphological and phylogenetic studies, *P.bambusicola* is introduced hereby as a new species occurring on bamboo in Yunnan, China.

#### ﻿ Arecophila

Taxon classificationFungiXylarialesCainiaceae

K.D. Hyde, Nova Hedwigia 63(1-2): 82 (1996)

0742922E-C1E2-5321-AB86-754C598280DC

MB27653

##### Notes.

The genus *Arecophila* is characterized by immersed ascomata, usually with a clypeus, unitunicate, cylindrical asci, commonly producing an apical ring, and ascospores with longitudinal striation or a verrucose wall, and surrounded by a mucilaginous sheath (Hyde 1996; Li et al. 2022). Li et al. (2022) provided a morphological comparison of the main characters of *Arecophila* species. The asexual morph of *Arecophila* has not been reported. According to Li et al. (2022), this genus is distributed across 12 countries and is reported from 16 host species.

#### ﻿ Arecophila xishuangbannaensis

Taxon classificationFungiXylarialesCainiaceae

L.S. Han & D.Q. Dai sp. nov.

59991F35-DF11-50B4-8D83-E6C9350C81E9

MB851853

Fig. 3

##### Etymology.

Named after the location “Xishuangbanna” where the new taxon was discovered.

##### Holotype.

GMB-W1283.

##### Description.

***Saprobic*** on dead culms of bamboo. **Sexual morph: *Ascomata*** 540–700 × 320–450 µm (x– = 586 × 389 µm, n = 20), immersed beneath a black clypeus, forming white ring surrounding ostioles of ascomata, solitary or scattered, sometimes gregarious, globose to subglobose, dark brown to black. ***Ostioles*** papillate, central, black. ***Peridium*** 15–25 µm thick, comprised of several layers, thick-walled, dense, brown to hyaline, cells of textura angularis. ***Paraphyses*** 2.5–6 μm wide, hyaline, numerous, cylindrical, unbranched, septate. ***Asci*** 180–270 × 12–14 μm (x– = 213 × 12.8 μm, n = 20), 8-spored, unitunicate, cylindrical, pedicellate, straight or slightly curved, apically rounded, with a 3.7–4.7 μm wide, 2.5–3 μm high (x– = 4.3 × 2.7 μm, n = 20), wedge-shaped, J+, apical ring. ***Ascospores*** 23–27 × 8.5–9.5 μm (x– = 24.5 × 8.8 μm, n = 20), overlapping, uniseriate, initially hyaline, pale brown to dark brown when mature, ellipsoidal, medianly 1-septate, tapering towards both ends, slightly constricted at the septum, with longitudinal striation along entire length of the ascospore, surrounded by a 3.5–5 µm thick, distinct, globose to subglobose, mucilaginous sheath. **Asexual morph**: Undetermined.

**Figure 3.**
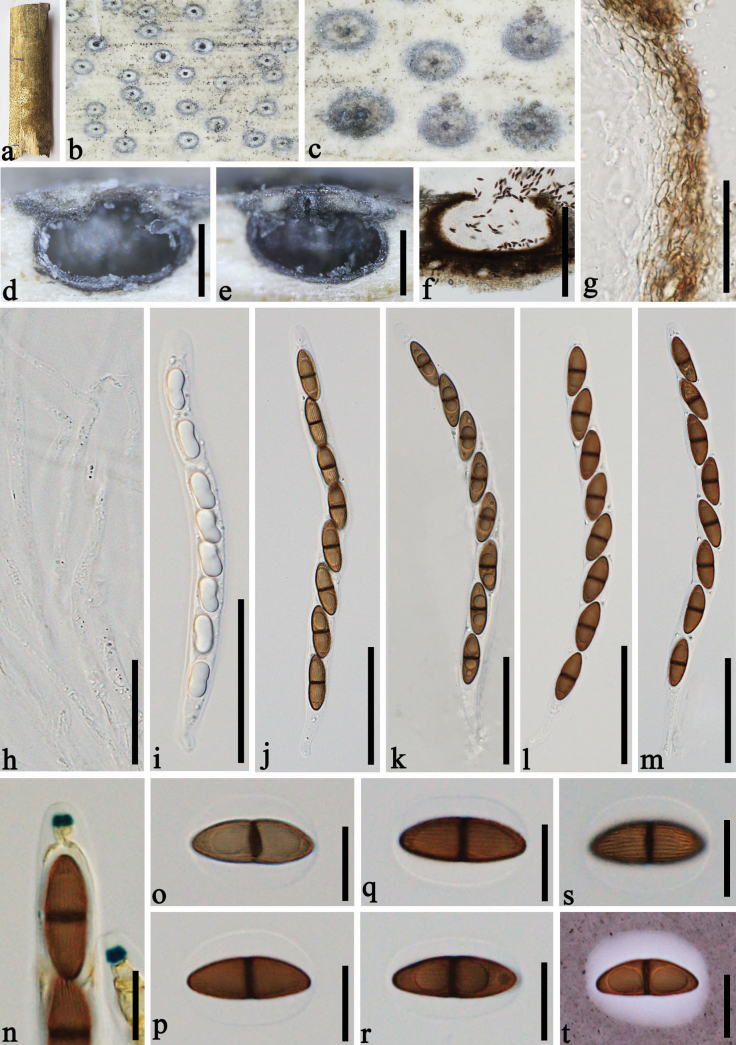
*Arecophilaxishuangbannaensis* (GMB-W1283, holotype) **a** bamboo specimen **b, c** appearance of ostioles on host surface **d–f** vertical sections of ascomata **g** peridium **h** paraphyses **i–m** asci **n** asci with J+, wedge-shaped rings (Stained in Melzer’s reagent) **o–t** ascospores (**s** showing ascospore with longitudinal striations **t** ascospore stained in Indian ink showing mucilaginous sheath). Scale bars: 300 μm (**d–f**); 20 μm (**g**); 30 μm (**h**); 50 μm (**i–m**); 15 μm (**n–t**).

##### Materials examined.

China, Yunnan Province, Xishuangbanna, Jinghong, Manzhang, Mengla, 21°91′97"N, 101°20′42"E, 617.14 m, on dead culms of bamboo, 16 August 2020, Dong-Qin Dai, Li-Su Han, DDQ00993, (GMB-W1283 holotype), *ibid*. (ZHKU 23-0258, isotype), *ibid*. DDQ00993-1 (ZHKU 23-0280).

##### Notes.

In the phylogenetic tree, our new collections of *Arecophilaxishuangbannaensis* (GMB-W1283, ZHKU 23-0280) formed a well-separated sister branch with *A.bambusae* (HKUCC 4794) and *Arecophila* sp. (HKUCC 6487) with 92% ML, 0.94 PP statistical supports (Fig. 1). Based on a nucleotide base pair comparison, *A.xishuangbannaensis* differs from *A.bambusae* (HKUCC 4794) in LSU gene (15/736 bp, 2%). Morphologically, *A.xishuangbannaensis* is similar to *A.bambusae*, in having cylindrical asci and ellispoidal ascospores. However, our new taxon differs *A.bambusae* by forming a white ring surrounding ostioles of ascomata and having larger asci (180–270 × 12–14 μm vs. 132.5–140 × 7.5–8 µm) and larger ascospores (23–27 × 8.5–9.5 μm vs. 19–22.5 × 5.5–7 µm) (Umali et al. 1999; Li et al. 2022). *Arecophilaxishuangbannaensis* also resembles *A.notabilis* K.D. Hyde, but it has larger ascomata (586 × 389 µm vs. 400 × 360 µm) (Hyde 1996). The spores of this species did not germinate on PDA or malt extract agar (MEA) media, thus no culture is available.

#### ﻿ Arecophila zhaotongensis

Taxon classificationFungiXylarialesCainiaceae

L.S. Han & D.Q. Dai sp. nov.

6E796CA0-45F3-5D29-907D-CA2900B11090

MB851836

Fig. 4

##### Etymology.

Named after the location “Zhaotong” where the new taxon was discovered.

##### Holotype.

GMB-W1353.

##### Description.

***Saprobic*** on dead culms of bamboo. **Sexual morph: *Ascomata*** 600–960 × 450–550 µm (x– = 710 × 500 µm, n = 20), immersed beneath blackened clypeus, clypeus well-developed, darkened raised discs, or as tiny ostiolar dots, solitary, scattered, sometimes gregarious, dark brown to black, globose to subglobose, papillate, with a central ostiole. ***Peridium*** 15–25 µm thick, comprising several layers, thick-walled, brown cells of textura angularis. ***Paraphyses*** 1–3 µm wide, hyaline, numerous, filiform, branched. ***Asci*** 190–240 × 10.5–14 µm (x– = 215 × 11.6 µm, n = 20), 4- or 8-spored, rarely 6-spored, cylindrical, unitunicate, short pedicellate, straight or slightly curved, rounded at the apex, with a 4–4.5 μm wide, 2–2.5 µm high (x– = 4.2 × 2.2 µm, n = 20), trapezoidal, J+, apical ring. ***Ascospores*** 21–30 × 6–8 µm (x– = 25.5 × 7 µm, n = 20), uniseriate or overlapping uniseriate, brown, ellipsoidal, 1-septate, septate at the centre, slightly tapering at the ends, with longitudinal and sulcate striations, surrounded by a 5–10.5 µm wide, distinct, oval to spherical, mucilaginous sheath. **Asexual morph**: Undetermined.

##### Culture characters.

Ascospores germinating within 24 h. Colonies reach 20 mm diam. in 15 days under dark and at 28 °C conditions, circular, hairy, white from above, and yellow to yellowish from below.

**Figure 4.**
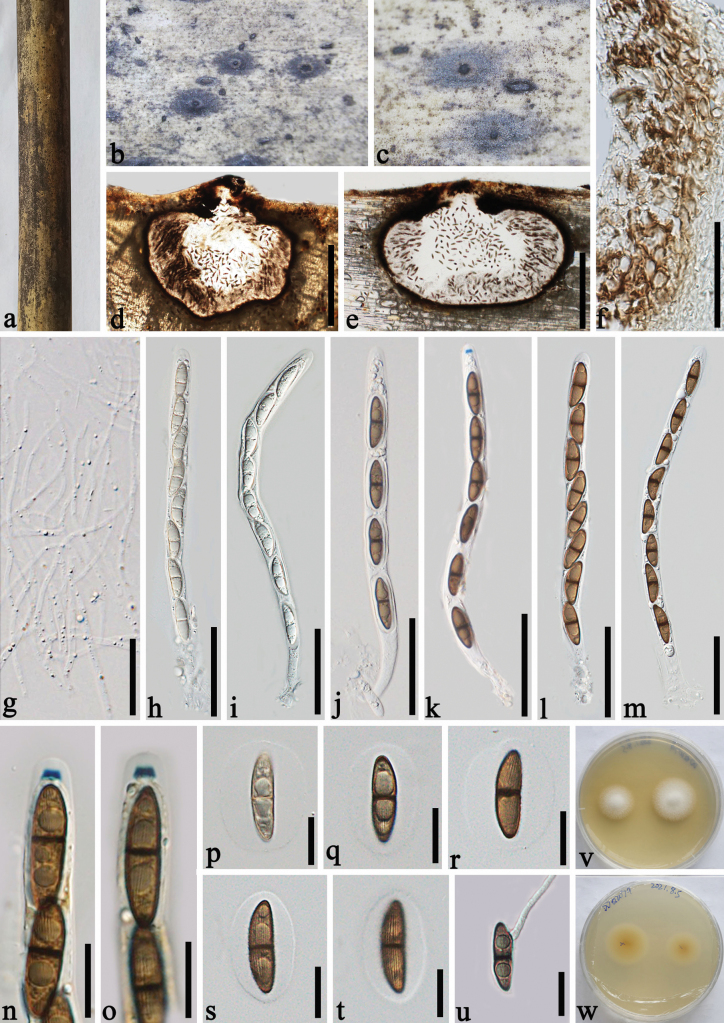
*Arecophilazhaotongensis* (GMB-W1353, holotype) **a** bamboo specimen **b, c** appearance of ostioles at the host surface **d, e** vertical sections of ascomata with ostioles and black clypei **f** peridium **g** paraphyses **h–m** asci **n, o** asci with a J+ trapezoidal ring (stained in Melzer’s reagent) **p–t** ascospores surrounded by mucilaginous sheath (**t** ascospore with longitudinal striations) **u** a germinating ascospore **v, w** cultures on PDA after 15 days (**v** upper, **w** reverse). Scale bars: 300 µm (**d, e**); 30 µm (**f, g**); 50 µm (**h–m**); 15 µm (**n–u**).

##### Materials examined.

China, Yunnan Province, Diqin, Shangri-La, Bigu Mountain, on dead culms of bamboo, 22 July 2020, 27°36′56.9"N, 99°42′6.4"E, 3460 m, Dong-Qin Dai DDQ00740 (ZHKU 23-0261); Zhaotong, Zhenxiong S302, 27°36′8"N, 104°56′34"E, 1673.07 m, on dead culms of bamboo, 29 July 2021, Dong-Qin Dai, Li-Su Han, DDQ02079, (GMB-W1353, holotype), GMBCC1145, ex-type; *ibid*. (ZHKU 23-0259, isotype), ZHKUCC 23-0975, ex-isotype; *ibid*. DDQ02105 (ZHKU 23-0260).

##### Notes.

In the phylogenetic tree, the new species *A.zhaotongensis* (GMBCC 1145, ZHKU 23-0259, ZHKU 23-0260) formed a separated sister branch to *A.bambusae* (HKUCC 4794), *Arecophila* sp. (HKUCC 6487) and *A.xishuangbannaensis* (GMB-W1283, ZHKU 23-0280) with 89% ML, 0.99 PP statistical supports (Fig. 1). Based on a nucleotide pairwise comparison, *A.zhaotongensis* differs from *A.bambusae* (HKUCC 4794) in 26/736 bp of LSU (3.5%), and differs from *A.xishuangbannaensis* (GMB-W1283, ZHKU 22-0280) in 56/563 bp of ITS (9.9%), 18/736 bp of LSU (2.4%). *Arecophilazhaotongensis* has larger asci than *A.bambusae* (190–240 × 10.5–14 µm vs. 132.5–140 × 7.5–8 µm) and larger ascospores (21–30 × 6–8 µm vs. 19–22.5 × 5.5–7 µm) (Umali et al. 1999). *Arecophilazhaotongensis* differs from *A.xishuangbannaensis* (GMB-W1283, ZHKU 23-0280) in having narrower ascospores (21–30 × 6–8 µm vs. 23–27 × 8.5–9.5 µm). The new species also resembles *A.muroiana* (I. Hino & Katum.) You Z. Wang et al. (Wang et al. 2004). However, *A.muroiana* lacks a clypeus absent, while a blackened clypeus was observed in *A.zhaotongensis*.

## ﻿Discussion

*Paramphibambusa* forms deeply immersed, dark ascomata, with a long neck, J+ asci and smooth-walled ascospores. Interestingly, genera in Cainiaceae usually form ascospores with longitudinal striations or germ slits or germ pores, however, these characters were not observed in our new collection (GMB-W1350). Hence, we introduced the new genus *Paramphibambusa* in Cainiaceae based on morphological characteristics and phylogenetic analyses (Fig. 1). Moreover, we introduced two new *Arecophila* species in Cainiaceae. The establishment of *Paramphibambusa* and the introduction of two new *Arecophila* species enriches the species diversity of the family Cainiaceae and the diversity of bambusicolous fungi.

Currently, some species in the Cainiaceae are monospecific, such as *Longiappendispora* (Mapook et al. 2020), and *Paramphibambusa* (this study), while *Amphibambusa*, and *Atrotorquata* each contain only two species (Kohlmeyer and Volkmann-Kohlmeyer 1993; Liu et al. 2015; Jiang et al. 2021). Hence, more samples are needed to better understand each genus. Wijayawardene et al. (2022b) mentioned that it is essential to carry out more studies on host plants (that have been extensively studied for fungi, such as bamboo) in biodiversity-rich regions to reveal more novel species. Yunnan is exceedingly rich in fungal diversity, especially in higher level taxa, such as ascomycetes and basidiomycetes (Wijayawardene et al. 2021b; Dai et al. 2022). Hence, we believe that future studies on bamboo-associated fungi in Yunnan Province would disclose more novel taxa.

*Atrotorquata* was introduced as a monotypic genus by Kohlmeyer and Volkmann-Kohlmeyer (1993) to accommodate *A.lineata* Kohlm. & Volkm.-Kohlm. Subsequently, Liu et al. (2015) introduced *A.spartii* Thambug et al. as the second species. These two species share similar morphology, but their phylogenetic relationship was not well-resolved by Liu et al. (2015). Due to a lack of sequence data in GenBank, *Atrotorquata* clusters outside of Cainiaceae. More sequences especially protein genes loci are needed, to clarify its family placement.

Eighteen epithets were listed in *Arecophila* (Li et al. 2022), but only four taxa and a unnamed species have available molecular data, *viz.*, *A.australis* Q.R. Li et al. (GZUCC0112, GZUCC0124), *A.bambusae* (HKUCC 4794), *A.clypeata* Q.R. Li et al. (GZUCC0110, GZUCC0127), *A.miscanthi* Q.R Li & J.C. Kang (GZUCC0122, MFLU 19-2333), and *Arecophila* sp. (HKUCC 6487). Thus, it is necessary to recollect fresh specimens and designate epitypes or reference specimens. Li et al. (2022) divided *Arecophila* into two clades based on phylogenetic analyses. We obtained the same results in our study, probably because most species of *Arecophila* lack protein genes regions in GenBank. We may need to design more suitable primers for sequencing protein genes fragments of *Arecophila* to support phylogenetic study.

## Supplementary Material

XML Treatment for Paramphibambusa

XML Treatment for Paramphibambusa bambusicola

XML Treatment for Arecophila

XML Treatment for Arecophila xishuangbannaensis

XML Treatment for Arecophila zhaotongensis

## ﻿Additional information

### Author ORCIDs

Li-Su Han https://orcid.org/0000-0001-5380-9928

Nalin N. Wijayawardene https://orcid.org/0000-0003-0522-5498

Chao Liu https://orcid.org/0000-0001-6811-2218

Li-Hong Han https://orcid.org/0000-0002-6127-0915

Itthayakorn Promputtha https://orcid.org/0000-0003-3376-4376

Qiang Li https://orcid.org/0000-0002-9735-8214

Abdallah M. Elgorban https://orcid.org/0000-0003-3664-7853

Salim Al-Rejaie https://orcid.org/0000-0002-9254-1087

Kazuaki Tanaka https://orcid.org/0000-0002-7037-0774

Dong-Qin Dai https://orcid.org/0000-0001-8935-8807

### Data availability

All of the data that support the findings of this study are available in the main text.
